# Evaluation of ambulance blood cultures in patients with suspected sepsis. A rural prospective cohort study

**DOI:** 10.1007/s15010-026-02830-x

**Published:** 2026-05-22

**Authors:** Lars-Jøran Andersson, Gunnar Skov Simonsen, Erik Solligård, Knut Fredriksen

**Affiliations:** 1 Department of Clinical Medicine, Anaesthesia and Critical Care Research Group, Faculty of Health Sciences, UiT The Arctic University of Norway, Tromsø, Norway; 2 Division of Emergency Medical Services, University Hospital of North Norway, Tromsø, Norway; 3 Department of Microbiology and Infection Control, University Hospital of North Norway, Tromsø, Norway; 4 Research Group for Host-Microbe Interaction, Faculty of Health Sciences, UiT The Arctic University of Norway, Tromsø, Norway; 5 Mid-Norway Sepsis Research Group, Department of Circulation and Medical Imaging, Norwegian University of Science and Technology, Trondheim, Norway; 6 Clinic of Anesthesia, St Olavs Hospital, Trondheim University Hospital, Trondheim, Norway

**Keywords:** Sepsis, Antibiotics, Blood cultures, Prehospital, Ambulance

## Abstract

**Purpose:**

To describe the epidemiology of blood cultures (BCs) drawn from patients with suspected sepsis by a rural ambulance service.

**Methods:**

Patients were included if they had clinically suspected sepsis and at least one BC was drawn. Variables associated with positive BCs were identified with logistic regression, culture results before and after ambulance antimicrobial therapy were compared and the susceptibility to empirical antimicrobial therapy in bacterial isolates was determined.

**Results:**

Among 392 included patients, sepsis severity score were higher when BCs were positive than negative, but there was no significant difference in 30-day all-cause mortality between BC negative and positive patients. In 47 patients ambulance blood cultures were positive (47/347 (13,5%)). Fever (OR 1.54, 95% CI 1.11–2.15) was associated with higher odds of positive cultures, while lower respiratory tract infection was associated with lower odds of positive cultures (OR 0.10, 95% CI 0.04–0.30). In 205 patients who had both ambulance and hospital BCs drawn and received ambulance antimicrobial therapy, only the ambulance BCs were positive in 16 patients, whereas in 4 patients only hospital cultures were positive. Bacterial isolates from positive BCs were susceptible to the empirical antimicrobial regimen in 80% of cases.

**Conclusion:**

Positive ambulance BCs were associated with higher sepsis severity, and ambulance cultures were most useful in febrile patients with a suspected focus of infection other than the lower airways. Repeated BCs after hospital admission added only limited diagnostic value if an adequate ambulance BC had already been drawn.

## Introduction

Sepsis is a life-threatening condition [1], and early diagnosis and therapy may decrease both morbidity and mortality [2–4]. Administration of intravenous antimicrobial therapy in the prehospital setting can improve survival from sepsis [5, 6]. Drawing of BCs before initiation of antimicrobial treatment is considered a cornerstone in sepsis treatment, since a positive culture may guide later selection of therapy against the causative microorganism and shorten the period of broad-spectrum empirical regimens [7]. However, current BC practices yield positive cultures in as few as 11.4% of the samples drawn in the emergency department [8], and this seriously limits the diagnostic value of the procedure. Sampling of BCs in the prehospital setting (ambulance BCs) poses additional challenges. Ideally, the sensitivity and specificity of ambulance BCs should be comparable to BCs drawn after arrival in hospital (hospital BCs), but the risk of BC contamination may be increased in the prehospital setting, and the increase in time until incubation may potentially lower the sensitivity [8]. Furthermore, drawing of BCs before transport may increase on-scene time and thus delay more necessary hospital diagnostics and treatment. These objections must be weighed against the potential value of a positive BC drawn before antimicrobial therapy is initiated. Finally, associations have been shown between focus of infection, disease severity [9] and time from antimicrobial therapy to hospital BCs [8, 10] and the probability of drawing positive BCs. Thus, a more selective and efficient BC strategy is possible.

High quality prehospital BC strategies have been shown to be feasible [5, 11–13], but the diagnostic value of the practice is still uncertain. It is not clear to what extent ambulance antimicrobial therapy renders hospital BCs negative. There is no single standard recommendation concerning ambulance BCs. The most common recommendation is to draw two sets of BCs before starting intravenous antimicrobial therapy. If the potential benefit for the patient is high, antimicrobial treatment can be started prior to BCs if they cannot be obtained in a timely manner [14]. In cases where drawing of BCs proves difficult, it is useful for the prehospital clinician to know if a BC is likely to be positive. Factors associated with positive ambulance BCs have not been described earlier. Furthermore, it is unclear how much ambulance BC sampling reduces the benefit of hospital BCs.

In the present study, the aim is to describe the ambulance BC epidemiology in an ambulance service with a standardised protocol for BCs and early administration of antimicrobial therapy in patients with suspected sepsis. We aim to examine the diagnostic value of drawing BCs before ambulance antimicrobial therapy and repeated BCs drawn in the hospital. Furthermore, we examine the association between positive BCs and sepsis severity represented by Sequential Organ Failure Assessment (SOFA)-score and factors associated with positive BCs that can be identified by ambulance personnel. Finally, we evaluate how well the empirical antimicrobial protocol covered the microbes detected in positive BCs.

## Methods

### Study setting and inclusion criteria

The study was conducted in the catchment area of the University Hospital of North-Norway (193 066 inhabitants/30 000 km^2^) with 29 ambulance stations and approximately 300 ambulance personnel. Patients with suspected sepsis managed by the ambulance service between May 2018 and August 2022 were prospectively included in a local quality register. The personnel were trained and used the clinical decision support tools quick Sequential Organ Failure Assessment (qSOFA) score, Systemic Inflammatory Response Syndrome (SIRS) score and Rapid Emergency Triage and Treatment System (RETTS) score [15]. Suspicion of sepsis was based on sepsis-3 definition [1] and clinical presentation, and patients did not need to fulfil scoring system criteria to be included in the register. In the present study we included patients from the register who were admitted to hospital and had ambulance and/or hospital BCs drawn during the first 72 h in hospital. Either a primary care physician or a physician at the closest hospital was consulted before antimicrobial therapy was started. Therapy was administered according to national guidelines (either penicillin + gentamicin, ampicillin + gentamicin, or cefotaxime) [16]. Prehospital data were recorded in real-time during the ambulance mission with the online tool REDCap (Research Electronic Data Capture, Paul Harris, Vanderbilt University Medical Center, 1211 Medical Center Drive, Nashville, TN 37232, USA). In-hospital data were retrospectively added to the register from the electronic patient management system DIPS (DIPS, 8006 Bodø, Norway).

### Treatment

All ambulance personnel received 4–6 h of training in sepsis management prior to the study. A low number of sepsis patients per personnel was expected, and since BC sampling was identified as a complex procedure, training was supported with an instruction video produced for this purpose [17]. Venous access was achieved either with a butterfly cannula (BD Butterfly needle, Beckton, Dickinson and Company, Singapore) or by a closed peripheral venous catheter (BD Nexiva^®^, Beckton, Dickinson and Company, Singapore). One set of BCs was defined as one aerobic and one anaerobic BACT/ALERT^®^ bottle (BioMerieux, Durham. NC, USA). Two sets were drawn whenever possible and the sets were drawn from two separate venous punctures, preferably one in each arm. BCs were stored in a paper envelope during transport and followed the patient to hospital in the ambulance. Children between 7 and 14 years had one BC set drawn, while in children under 7 years only a single pediatric aerobic BACT/ALERT^®^ bottle (BioMerieux, Durham. NC, USA) was used.

### Variables

The study included ambulance and hospital BCs. BCs were classified as positive if they contained a probable causative microorganism. Single BCs were classified as contaminated if they contained typical skin bacteria and there were no indwelling catheters or history of recent surgery. These BCs were categorized as negative for relevant pathogens. Patients with two or more BCs with growth of the same skin bacteria were defined as positive.

Clinical variables included standard measurements (oxygen saturation, respiratory rate, heart rate, systolic blood pressure, diastolic blood pressure, Glasgow coma score and body temperature), the presence of marbled, ashen or cyanotic skin colour, the assumed focus of infection and nursing home status were documented by ambulance personnel. NEWS2 was calculated retrospectively based on values registered by ambulance personnel, and Charlson comorbidity index score was calculated based on information from admission records. The SOFA score was determined from in-hospital records, and 30-day all-cause mortality was retrieved from the Norwegian National Population Register. The assessment of antimicrobial coverage was based on in vitro susceptibility data from the detected bloodstream isolates and antimicrobial treatment given in the prehospital setting and did not consider dosage or host parameters such as kidney function or focus of infection.

### Analysis

Patient and management data from the study are presented with median values and interquartile ranges (IQR) for continuous data and with absolute values and percentages for dichotomous data. Proportions of positive BCs are presented in percent with 95% confidence intervals (CI). Student t-test was used to examine differences in SOFA score and Wald test for differences in 30-day all-cause mortality between patients with negative and positive BCs. Logistic regression analysis was used to find variables that can be recorded in the prehospital setting and were associated with positive ambulance BCs. With 15 predictor variables and nearly 400 patients the study was large enough for regression analysis; however, the sample size was not sufficient to analyse data within the group of patients with positive BCs. Missing vital parameters (Table 1) were managed by multiple imputation using five imputations. We used backward selection and variables with p-values < 0.05 were considered significant. The model was adjusted for age and sex. Results from the regression analysis are presented with odds ratios and 95% CIs. A Sankey-diagram and a grouped bar diagram was used to describe sampling, antimicrobial treatment and susceptibility to empirical antimicrobial treatment of the positive BCs, respectively. All analyses were performed using SPSS version 29.0.1.0 (IBM Corp, Armonk, NY, USA).

**Table 1:** Patient and management characteristics for all patients and stratified by positive or negative blood cultures (BCs)

Variable	All *N* = 392	Negative BC *N* = 331	Positive BC *N* = 61
Age (Years)	76 (65, 84)	77 (65, 84)	74 (66, 82)
Male	196 (50.0%)	165 (49.8%)	31 (50.8%)
Female	196 (50.0%)	166 (50.2%)	30 (49.2%)
Systolic blood pressure (mmHg)	121 (102 ,141)	121 (103, 142)	121 (98, 140)
Diastolic blood pressure (mmHg)	70 (60, 81)	70 (60, 81)	70 (59, 80)
Temperature (˚C)	38,4 (37.5, 39.0)	38.3 (37.5, 38.9)	38.8 (38.1, 39.5)
Respiratory rate (breaths/min)	28 (24, 30)	28 (24, 32)	26 (22, 30)
Heart rate (beats/min)	105 (90, 120)	105 (90, 120)	105 (95, 114)
Glascow Coma score	15(14, 15)	15 (14, 15)	15 (14, 15)
Oxygen saturation (%)	93 (90, 95)	93 (89, 95)	94 (92, 96)
National early warning score 2	10 (7, 12)	9 (7, 12)	10 (7, 12)
Marbled or ashen skin	68 (17.3%)	54 (16.3%)	14 (23.0%)
Cyanotic skin, lip or tounge	34 (8.7%)	34 (10.3%)	0 (0.0%)
Assumed focus of infection lower airways	152 (38.8%)	148 (44.7%)	4 (6.6%)
Assumed focus of infection urinary tract	118 (30.1%)	94 (28.4%)	24 (39.3%)
Nursing home resident	92 (23.5%)	83 (25.1%)	9 (14.8%)
Charlson comorbidity score	5 (3,7)	5 (3, 7)	5 (3, 7)
Sequential organ failure assessment-score	2 (1, 3)	2 (1, 3)	2 (1, 5)
Septic shock after hospital arrival	29 (8.7%)	17 (5.1%)	12 (19.7%)
30-day all-cause mortality	40 (10.2%)	35 (10.6%)	5 (8.2%)
Prehospital antimicrobial treatment	324 (82.7%)	275 (83.1%)	49 (80.3%)

Continuous variables are given with median values and interquartile range. Dichotomous variables are given with absolute values and percent

## Results

**Fig. 1:**
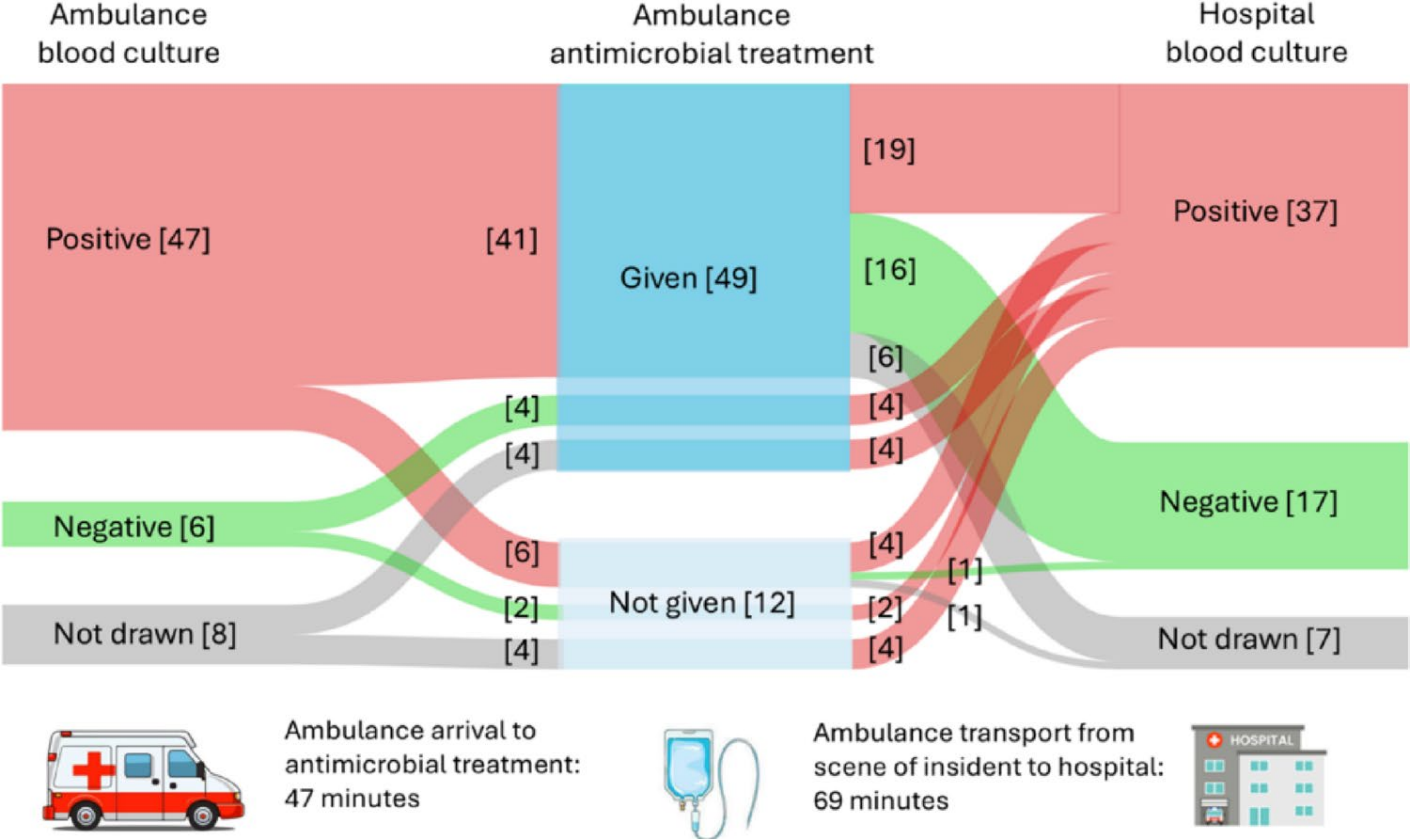
Overview of 61 patients with sepsis suspected in the prehospital setting and positive blood cultures (BCs) from ambulance sampling and/or within the first 72 h of hospital stay. Number of patients in brackets. The left column shows number of patients with positive BCs from ambulance sampling. Ambulance BCs were drawn from 347 patients. The column in the middle shows whether the patients were among the 324 patients that received ambulance antimicrobial treatment. The column to the right shows number of patients with positive BCs from hospital sampling. Hospital BCs were drawn from 283 patients. The ambulances arrived 19 min after call was received by the emergency dispatch centre, antimicrobial treatment was started 47 min after ambulance arrival when the ambulance started transport towards hospital. Transport time to hospital was 69 min, and time from call to arrival at hospital was 135 min (all time intervals are median values for the 49 patients that received prehospital antimicrobial treatment)

From the ambulance register 413 patients were eligible for inclusion, but 7 did not consent, 8 were not admitted to hospital and BCs were not drawn in 6 patients. Thus, 392 patients with BC’s drawn either in the ambulance (347), and/or after admission to hospital (283) were included in the study. Ambulance antimicrobial treatment was started in 324 patients. At least one BC was positive in 61 patients, bacteria from positive BCs are described in additional table A. PCR was positive for 42 patients. An overview of BCs and antimicrobial treatment started in the ambulance in patients with positive BCs is given in Fig. 1. Probable contaminants were detected in 23 of 468 ambulance BCs (4.9%).

### Description of the study group

Patient and management characteristics are presented in Table 1. Respiratory rate, heart rate and body temperature were higher, and oxygen saturation was lower than normal values, while blood pressure was within normal values. Lower airways were the assumed focus of infection in 152 (38.8%) patients, and 30-day all-cause mortality was 10.2%.

### Patients with positive versus patients with negative BCs

Sepsis severity as defined by SOFA score was significantly higher in patients with a positive BC than in patients with negative BCs (mean SOFA score 2.9 vs. 2.2, *p* = 0.019). The 30-day all-cause mortality was not significantly different between these two groups, with 8.2% mortality among patients with positive BCs vs. 10.2% among patients with negative BCs (*p* = 0.57).

### Factors associated with positive ambulance BCs

At least one positive ambulance BC was found in 47 of 347 (13.5%) patients. Body temperature (OR 1.54, 95% CI 1.11–2.15) was independently associated with a higher proportion of positive ambulance BCs, whereas lower airways as assumed focus of infection was independently associated with a lower proportion (OR 0.10, 95% CI 0.04–0.30). Unadjusted ORs are given in Table 2. The assumed focus of infection in the patients with positive ambulance BCs is presented in Table 3.

**Table 2:** Logistic regression analysis of variables associated with positive ambulance blood culture

Variable	All, *N* = 347	Unadjusted OR	Adjusted OR
Age (Y)	76 (64, 84)	1.01 (0.99, 1.03)	1.02 (1.00, 1.04)
Male	171 (49.3%)	1.12 (0.61, 2.08)	1.02 (0.53, 1.96)
Female	176 (50.7%)		
Systolic BP (mmHg)	124 (10 ,143)	1.00 (0.99, 1.01)	
Diastolic BP (mmHg)	70 (60, 81)	1.00 (0.98, 1.02)	
Temperature (Celcius)	38,4 (37.6, 39.0)	**1.46 (1.07**,** 2.00)**	**1.54 (1.11**,** 2.15)**
Respiratory rate (breaths/min)	28 (24, 30)	**0.94 (0.90**,** 0.99)**	
Heart rate (beats/min)	105 (91, 120)	0.99 (0.98, 1.01)	
Glascow Coma score	15(14, 15)	0.85 (0.72, 1.01)	
SpO2 (%)	93 (90, 95)	1.08 (1.00, 1.17)	
Marbled or ashen skin	59 (17.0%)	1.38 (0.64, 2.97)	
Assumed focus of infection lower airways	138 (39.8%)	**0.12 (0.04**,** 0.33)**	**0.10 (0.04**,** 0.30)**
Assumed focus of infection urinary tract	107 (30.8%)	1.81 (0.96, 3.40)	
Charlson Comorbidity score	5 (3,6)	1.05 (0.94, 1.18)	

Continuous variables are presented with median values and interquartile range, and dichotomous variables are presented with absolute numbers and percent. Unadjusted odds ratios are presented for all variables, while adjusted odds ratios are shown for variables in the final model. All odds ratios are presented with 95% confidence intervals and significant values are written in bold letters

**Table 3:** Number of patients with positive ambulance blood cultures stratified by assumed focus of infection

Source of infection	Patients tested	Positive ambulance BC
All	347	47 (13.5%, CI 9.9–17.2)
Urinary tract	107	20 (18.7%, CI 11.3–26.1)
Lower airways	138	4 (2.9%, CI 0.0-5.7)
Skin	19	4 (21.1%, CI 2.7–39.4)
Abdomen	16	2 (12.5%, CI 0.0-28.7)
Unknown	66	12 (18.2%, CI 8.8–27.5)
Postoperative wound infection	11	2 (18.2%, CI 0.0-40.1)
Other focus	15	3 (20.0%, CI 0.0-40.2)

More than one assumed focus of infection was reported in 25 patients. Proportions are given in percent with 95% confidence intervals (CI). Positive ambulance BC was found in 43 (20.6%, CI 15.1–26.1) of 209 patients with focus other than lower airways. BC: blood culture

### BCs drawn before versus after ambulance antimicrobial treatment

In 205 cases ambulance antimicrobial treatment was given after ambulance BCs were drawn and before hospital BCs were drawn. In this group we found 39 patients with positive BCs. The ambulance BCs were positive in 35 patients (17.1%, 95% CI 11.5–22.7) and hospital BCs were positive in 23 patients (11.2%, 95% CI 6.9–15.5). In 16 patients, a positive BC was found only in the ambulance BCs, and in no more than 4 patients only the hospital BC was positive. Thus, drawing of 205 ambulance BCs resulted in 7.8% (95% CI 4.1–11.5) additional positive BCs that were not confirmed by a hospital BC in the same patient, and drawing of 205 hospital BC resulted in only 2.0% (95% CI 0.0- 3.8) additional positive BCs not already present in the ambulance BC.

### In-hospital BCs in patients with or without ambulance antimicrobial treatment

Hospital BCs were drawn from 283 patients. In this group, 228 patients had received antimicrobial treatment before blood sampling. Among these, we found 27/228 (11.8%, 95% CI 7.6–16.0) positive hospital BCs, and among patients that did not receive this treatment 10/55 (18.1%, 95% CI 8.0-28.4) had positive hospital BCs.

### Susceptibility to antimicrobial treatment

**Fig. 2:**
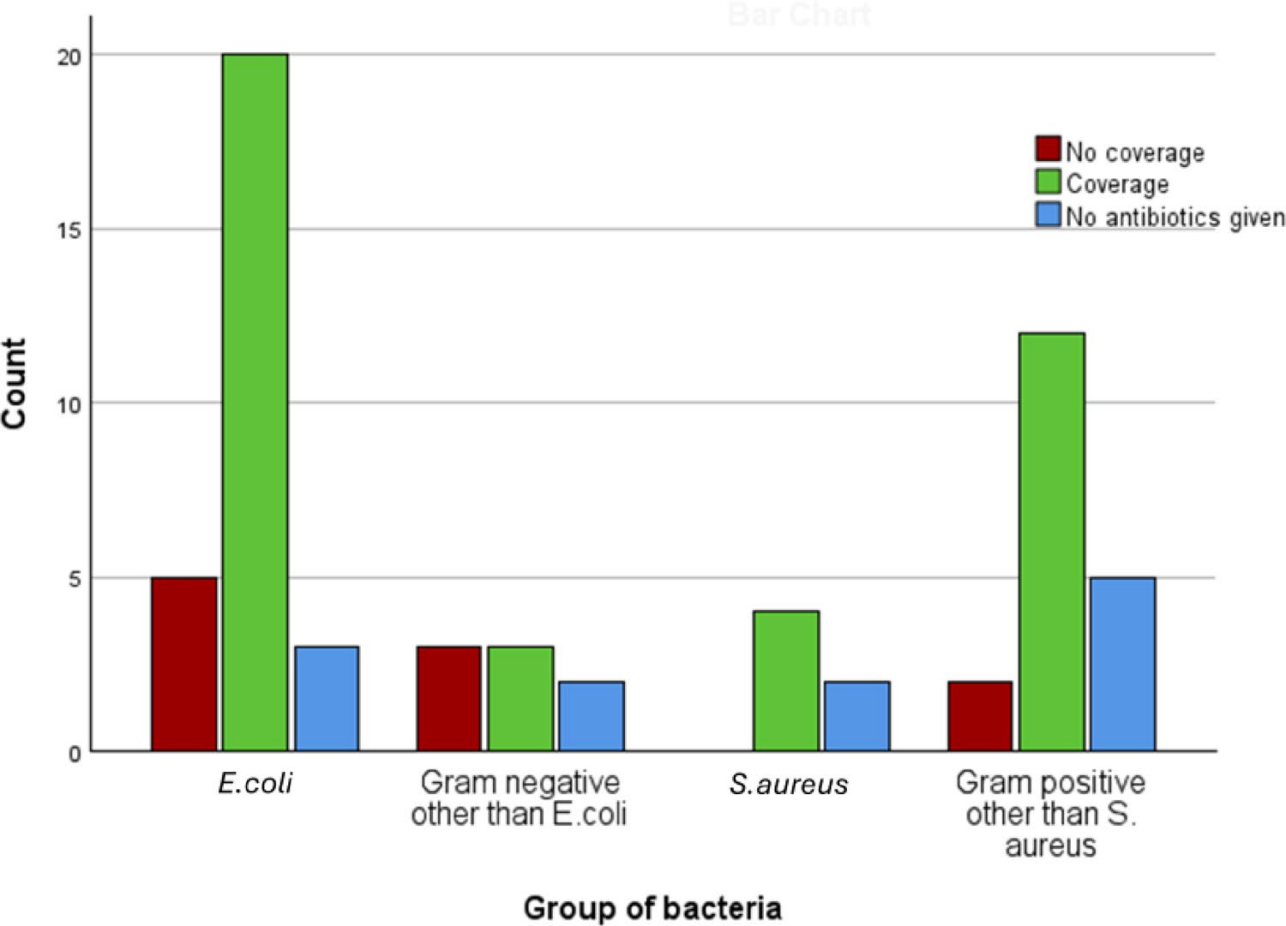
Susceptibility of blood culture isolates to the empirical antimicrobial therapy. The blood cultures were drawn either prehospitally or within 72 after arrival at hospital in 61 patients. Empirical antimicrobial therapy was started by the ambulance personnel. There was no coverage in 10 patients, but therapy covered the identified microbes in 39 patients. Twelve patients did not receive antimicrobial treatment by the ambulance.

Ambulance antimicrobial treatment was given to 49 of the patients with positive ambulance or hospital BCs. Seven patients had bloodstream isolates that were susceptible to two antimicrobial agents given in the ambulances, 32 had isolates susceptible to one agent and 10 received ambulance treatment that did not cover the detected microbes. In total, 39/49 (79.6%) were covered by the empirical antimicrobial protocol, including 33/40 (82.5%) of patients receiving a gentamicin-based regimen and 7/9 (77.8%) of patients receiving cefotaxime. One patient had coverage from both regimens. Among the 10 patients without antimicrobial coverage, one patient had no ambulance BC, and eight patients had positive ambulance BCs. The course of BCs in the cohort is shown in Fig. 2.

## Discussion

The main finding in this study was that ambulance sampling of BCs was feasible and of good quality. Time interval from call to arrival at hospital was 135 min and similar to a study of prehospital antimicrobials and BCs in Wales [18]. Furthermore, we identified prehospital variables that increased the probability for a positive BC, and we found that repeated BCs after hospital admission had only limited value. Sepsis severity correlated with a positive BC and the empirical antimicrobial regimen used covered 79.6% of the bacterial isolates.

The patients showed physiological measurements slightly outside normal values, as expected. The proportion of positive ambulance BCs (13.5%) was comparable to the results from the PHANTASi-trial [13]. The proportion of positive prehospital BCs from patients with suspected sepsis varies between 14 and 42% [11–13, 19], probably due to differences in study inclusion. The rate of contaminated BCs should be kept as low as possible, but the prehospital environment and the low frequency of procedures may increase the rate of contamination and false positive cultures. Some guidelines state that the rate should be lower than 3% [20], but reported rates range between 1 and 17% [8, 21, 22]. The contamination rate in ambulance BCs in the present study was low (4.9%) when compared to earlier studies of prehospital [11, 12] as well as in-hospital [8, 21, 22] BC sampling. This may be explained by the focused training of ambulance personnel and suggests that prehospital BCs do not differ from in-hospital samples when it comes to quality.

We found no significant difference in the 30-day all-cause mortality between patients with and without positive BCs, but the sepsis severity score was significantly higher in the group with positive BCs. Other studies have also reported higher sepsis severity scores in patients with positive BCs [13, 23], but the literature is conflicting with regard to mortality [13, 23–25]. Sepsis score may also be a more relevant outcome than 30-day all-cause mortality, as the latter is strongly influenced by other factors than the infectious disease itself, like e.g. comorbidities. The mortality in patients with positive BCs in our study (8.2%) was close to the mortality reported in the PHANTASi-trial (9.6%) [13]. The inclusion of patients early in the course of disease leads to a lower mortality than in studies from hospitals, as reported recently from a Hungarian emergency department (38.6%) [26]. This illustrates that mortality in cohorts of sepsis patients with positive BCs varies, and may depend on the stage of the disease, which may be more advanced in a hospital cohort. The threshold for BC sampling might also have been lower in the present and the PHANTASi-trials [13].

A suspected focus of infection in the lower airways was associated with a low rate of positive BCs, and this corresponds to earlier studies that also report a higher proportion of culture-negative sepsis in patients with respiratory tract infections [13, 23]. In the present study, this may in part be explained by a high proportion of cases with viral infections among patients with suspected lower respiratory tract infections. The odds for positive BCs in patients with an infectious focus in the lower airway is low, and the usefulness of BCs in these cases is debatable. Depending on the distance to hospital and the clinical condition of the patient, postponing prehospital treatment and BC sampling may be considered in this group. The increased body temperature in patients with positive BCs is consistent with earlier studies [13].

A positive BC was found only in the ambulance BCs in 16 patients where antimicrobial treatment was given after the ambulance BCs were drawn. In comparison, only 4 patients in the same group had BCs only positive after hospital sampling, and not in the ambulance BCs. This suggests that repeating BCs after admission is of limited value when a prehospital BC has already been drawn. Hospital BCs were positive in 11.8% of patients who had received ambulance antimicrobial therapy, compared to 18.1% of those who had not received such treatment. However, the figures in our study were too small to show a statistically significant difference in this respect. If we had omitted ambulance BC sampling in this study, we would have missed 16 of 39 patients with positive BCs. We also compared the proportion of positive hospital BCs among patients who received ambulance antimicrobial treatment with patients who did not, but the number of patients was too low for testing. No previous study has reported the effect of prehospital intravenous antimicrobials on the yield of subsequent hospital BCs. Two studies from the Netherlands report the effect of peroral antimicrobial treatment on yield of emergency room BCs, but with conflicting results [8, 13]. The effect of intravenous antimicrobial treatment on subsequent BCs should be investigated in further studies.

The prehospital empirical antimicrobial regimens in this study were microbiologically effective in 79.6% of cases. Other prehospital studies with third generation cephalosporin or piperacillin/tazobactam regimens reported coverage in the range of 72–87% [12, 13, 27]. The selected regimens should preferably be active against all relevant pathogens, but this must be balanced against unnecessary ecological consequences of broad-spectrum antimicrobial therapy. In the prehospital setting the diagnosis will be uncertain in many cases, but the optimal prehospital antimicrobial coverage for patients with more serious conditions should be further investigated and discussed. The significant proportion of patients without empirical antibiotic coverage emphasizes the necessity of retrieving BCs for later adjustment of therapy.

To the best of our knowledge, this is the first analysis of BC epidemiology in an ambulance service with established antimicrobial treatment and routine BC sampling in patients with suspected sepsis. Data were collected from an ambulance quality register and recorded immediately after each ambulance mission. The data quality is therefore good, and better than for data retrospectively extracted from patient records. The study was set in a sparsely populated area. This gave us the opportunity to study BC sampling performed by health care professionals who seldom draw BCs.

The setting also made it more challenging to get a sufficient sample size. The sample size did not allow for analyses within the group of patients with positive blood cultures. We wanted to include time for prehospital BCs, but there were to many missing data, however ambulance personnel were instructed to draw BCs before administration of antimicrobial treatment. Very few patients below 14 years, and no children younger than 7 years were included in this study. The large discrepancies in mortality among studies of patients with positive BCs suggest that there is a difference in the threshold for BC sampling and admission to hospital.

## Conclusion

Prehospital sampling of BCs in a mainly rural ambulance service may be feasible and of good quality. The usefulness of BC sampling in the prehospital setting is higher in patients with a suspected focus of infection other than the lower airways. BC sampling after hospital arrival may be useful in some cases even if intravenous antimicrobial treatment is given before admission. However, we expect to find a limited number of new positive cultures when prehospital BC sampling is adequate.

## Data Availability

The datasets generated during and analysed during the current study are publicly available from the Open Research Data depository of the UiT - The Arctic University of Norway: Andersson, Lars-Jøran; Fredriksen, Knut; Solligård, Erik; Simonsen, Gunnar Skov, 2025, “Background Data for: Evaluation of ambulance blood cultures in patients with suspected sepsis. A rural prospective cohort study”, https://doi.org/10.18710/EKPTQX, DataverseNO, V1.
